# Disparity in the era of personalized medicine for epithelial ovarian cancer

**DOI:** 10.1177/17588359221148024

**Published:** 2023-01-10

**Authors:** Michael-John Devlin, Rowan E. Miller

**Affiliations:** Barts Cancer Institute, Queen Mary University of London, Charterhouse Square, London EC1M6BQ, UK; Department of Medical Oncology, St Bartholomew’s Hospital, London, UK; Department of Medical Oncology, St Bartholomew’s Hospital, London, UK; Department of Medical Oncology, University College London Hospital, London, UK

**Keywords:** clear-cell ovarian cancer, clinical trials, epithelial ovarian cancer, low-grade serous ovarian cancer, mucinous ovarian cancer, novel therapeutics

## Abstract

The treatment of high-grade serous ovarian cancer and high-grade endometrioid ovarian cancer has seen significant improvements in recent years, with BRCA1/2 and homologous recombination status guiding a personalized approach which has resulted in improved patient outcomes. However, for other epithelial ovarian cancer subtypes, first-line treatment remains unchanged from the platinum–paclitaxel trials of the early 2000s. In this review, we explore novel therapeutic approaches being adopted in the treatment of clear cell, mucinous, carcinosarcoma and low-grade serous ovarian cancer and the biological rational behind them. We discuss why such disparities exist, the challenges faced in conducting dedicated trials in these rarer histologies and look towards new approaches being adopted to overcome them.

## Introduction

Cancer treatment has undergone rapid advancements in recent years, with high-grade serous ovarian cancer (HGSOC) and high-grade endometrioid ovarian cancer (HGEOC) being the exemplars of this phenomenon in advanced epithelial ovarian cancer (EOC). SOLO1 demonstrated a significant reduction in disease progression or death in patients with HGSOC/HGEOC who harboured a *BRCA1/2* mutation and were treated with the poly-ADP ribose polymerase (PARP) inhibitor olaparib following first-line treatment.^1^ Following on from this, PAOLA-1 demonstrated that the maintenance combination of olaparib and bevacizumab [a humanized monoclonal IgG1 antibody targeting the vascular endothelial growth factor receptor (VEGFR) ligand] improved progression-free survival (PFS) for HGSOC/HGEOC that are homologous recombination deficient (HRD), either due to the presence of a *BRCA1/2* mutation and/or a high genomic stability score.^2^ No benefit was observed with the addition of olapairb to bevacizumab in the HRD test negative patients leading to EMA/FDA approval for HRD-positive EOC only. In contrast, in the PRIMA trial, a benefit from PARP inhibition with niraparib was observed in the HRD test negative patients, albeit to a lesser magnitude.^3^ This has resulted is a personalized treatment paradigm for first-line maintenance therapy based on *BRCA1/2* and HRD status, leading to improved outcomes for patients with HGSOC/HGEOC.

However, for other EOC subtypes, such as clear-cell ovarian cancer (CCOC), mucinous ovarian cancer (MOC), low-grade serous ovarian cancer (LGSOC) and ovarian carcinosarcoma (OCS), the current standard of care remains largely unchanged since the platinum–paclitaxel trials of the early 2000s,^4–6^ studies in which these pathologies were under represented. In this review, we will evaluate the emerging therapeutic approaches to the treatment of these histologies, which to date, have lagged behind in the personalized medicine revolution.

### Why such discrepancies exist

**Figure 1.**
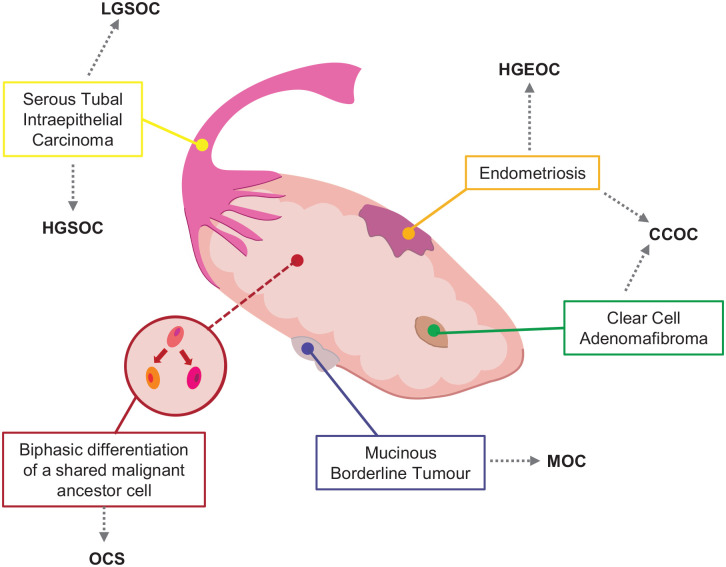
The genesis of different epithelial ovarian cancer histologies. Epithelial ovarian cancer comprises a number of distinct disease entities which arise from separate precursor lesions. Both high- and low-grade serous carcinomas are thought to arise from within the fallopian tubes; endometriosis has links to both HGEOC and clear-cell ovarian cancer, with clear cell also being associated with clear-cell adenofibromas in the absence of endometriosis. MCOs demonstrate a stepwise progression from benign ovarian tumours through to a mucinous borderline tumours. Ovarian carcinosarcomas are biphasic, with a high-grade carcinomatous and high-grade sarcomatous populations, thought to arise from a shared malignant ancestor cell. CCOC, clear-cell ovarian cancer; HGEOC, high-grade endometrioid ovarian cancer; HGSOC, high-grade serous ovarian cancer; LGSOC, low-grade serous ovarian cancer; MOC, mucinous ovarian cancer; OCS, ovarian carcinosarcoma.

It is important to appreciate why such discrepancies in personalized treatments exist as it highlights the challenges faced in the development of new therapeutics. As shown in Figure 1, rather than being a single pathology, EOC comprises a number of distinct disease entities which arise from separate precursor lesions, each one being molecularly unique.^7^ Both high- and low-grade serous carcinomas are thought to arise from within the fallopian tubes,^8–11^ whereas the pathogenesis of CCOC has links to both endometriosis and, less frequently, clear-cell adenofibromas.^12^ Mucinous ovarian cancers (MOCs) demonstrate a stepwise progression from benign ovarian tumours through to a mucinous borderline tumours and eventually MOC.^13^ OCS are biphasic, with a high-grade carcinomatous and high-grade sarcomatous populations, thought to arise from a shared malignant ancestor cell.^14^ The majority of OCS harbour an epithelial component of HGSOC (79.3%), with the remaining being endometrioid. Within the sarcomatous compartments, the pathology can either be homologous or heterologous, with chondrosarcoma and rhabdomyosarcoma being the most common.^14^ The clinical consequences of such diversity can be observed in our current standard of care treatments, with CCOC, LGSOC, OCS and MOC displaying a significantly lower response rate and PFS to platinum–paclitaxel compared to HGSOC/HGEOC.^15,16^

Compounding this histological and molecular diversity is the frequency and FIGO stage that each pathology presents with. HGSOC is the most common subtype, accounting for 80% of advanced EOC cases. This results in a large population of patients with advanced disease who can enter into clinical trials, particularly when compared to other histologies which are much less common and are more likely to present at an early stage. In addition, HGSOC patients survive longer, often allowing for entry into multiple clinical studies, whereas the overall survival (OS) of advanced non-HGSOC pathologies such as CCOC is significantly shorter,^17^ which may be due to the innate biology of the disease or due to the current lack of effective first-line treatments. The cumulative result is that it is more difficult to run clinical trials for rarer histologies. This was illustrated in the GOG0241 trial, a phase III study which aimed to explore the use of colorectal chemotherapy regimens in the first-line treatment of MOC, which closed early due to slow accural^18^ and in the GOG study which explored the role of cisplatin in OCS, and took over 20 years to recruit 136 patients.^19^

## Clear-cell ovarian cancer

ARID1A (the AT-rich interaction domain 1A, BAF250) is a sub-unit of the SWItch/Sucrose Non-Fermentable (SWI/SNF) chromatin remodelling complex and mutated in up to 67% of patients with CCOC.^20^ While ARID1A is the most frequently mutated subunit of the SWI/SNF complex, other mutations in this complex also occur; ARID1B, which is mutually exclusive with ARID1A, is mutated in 10–18% of CCOC^21,22^; SMARCA2 and SMARCA4 are mutated in 2%^22^ and 6.25%,^23^ respectively, and SMARCC1 is mutated in 2% of CCOC.^22^

The phosphatidylinositol 3-kinase (PI3K)-AKT pathway is the most frequently dysregulated pathway in human cancer. This pathway is activated in response to insulin, growth factors and cytokines and regulates key cellular processes such as metabolism, motility, growth and proliferation.^24,25^ In CCOC, mutations can occur at multiple points in this pathway. PIK3CA, which codes for the PI3K catalytic subunit (p110) is mutated in 20–50% of CCOC,^20–22,26–30^ while PIK3R1 which encodes the regulatory subunit (p85) is mutated in 7%. On whole genome sequencing, phosphatase and tensin (PTEN) homolog mutation was noted in 2% of cases in a Japanese study,^22^ while whole exome sequencing of a Korean cohort found mutations in 13.3% of cases.^27^ Mutations of AKT1 and AKT2 were found in 4% and 9% of cases, respectively.^22^ The PI3K pathway is interconnected with the MAP kinase pathway through RAS proteins, which can interact directly with the catalytic structure of PI3K. Kirsten rat sarcoma viral oncogene homologue (KRAS), one of the three RAS protein homologues, is mutated in up to 20% of CCOC.^27^

### Molecular-guided therapies

In a pre-clinical study, loss of ARID1A resulted in topoisomerase 2A and cell-cycle defects, which subsequently caused an increased reliance on the Ataxia-Telangiectasia Mutated and Rad3-related protein kinase (ATR). By inhibiting ATR in ARID1A-deficient cell lines, cell death occurred due to premature mitotic entry and genomic instability.^31^ The clinical trial ATARI^32^ is currently exploring the efficacy of the ATR inhibitor AZD6738 either as monotherapy or in combination with the olaparib in patients with ARID1A mutant clear-cell gynaecological malignancies.

A series of high-throughput cell-based drug screens in ARID1A mutant CCOC identified sensitivity to the YES1 kinase inhibitor dasatinib, which may be due to the drug reversal of p21 dysregulation that is caused by ARID1A deficiency.^33^ There has been no CCOC-specific studies exploring this therapeutic approach; however, two patients with CCOC were included in a phase II study of dasatinib in recurrent EOC; although histology-specific outcomes were not given, this study reported no objective responses.^34^

Chromatin is made more accessible by two major mechanisms; displacement of histones (the major proteins in chromatin) by chromatin remodelling complexes like SWI/SNF or by the modification of these histones by the addition of acetyl, methyl or phosphate groups, with histone deacetylases (HDAC) being critical to histone acetylation and deacetylation.^35^ Inhibition of HDAC6 promotes apoptosis in ARID1A mutant cell lines in 2D and improved survival in nude mice with orthotopically transplanted CCOC. HDAC inhibition also reduced tumour size in a genetic mouse model of CCOC.^36^ The combination of carboplatin with the HDAC inhibitor belinostat in platinum-resistant EOC saw a response rate of 7.4% (2/27) in a phase II study and did not proceed to a larger study.^37^ Another HDAC inhibitor, entinostat, in combination with olaparib was being evaluated in a phase I/II study^38^ which included patients with clear-cell gynaecological malignancies; however, this was terminated early in 2022 having enrolled 3 patients of a planned 73.

### Targeting angiogenesis

Angiogenesis plays a central role in the tumour development of EOC with pre-clinical studies demonstrating that inhibition of VEGF can stall tumour progression.

Bevacizumab is a recombinant humanized monoclonal IgG1 antibody that targets VEGFR ligand and is licenced for the treatment of EOC.^39,40^ Two phase III trials explored the addition of bevacizumab to carboplatin and paclitaxel as first-line treatment of EOC. GOG218^41^ used a treatment dose of bevacizumab of 15 mg/kg while ICON7,^42^ dosed at 7.5 mg/kg. Both studies reported a modest improvement in PFS with the addition of bevacizumab (GOG218; 4.1 months, ICON7; 1.5 months), with no improvement in OS. Neither study reported CCOC-specific outcomes. In the prospective observational study JGOG3022,^43^ bevacizumab was administered at 15 mg/kg and started concurrently with carboplatin–paclitaxel. Within this study, 36/293 patients had CCOC, 11 of which had measurable disease. Using RECIST 1.1,^44^ the response rate to treatment was 63.6%. The median PFS for CCOC of 12.3 months (8.3–15.3) was lower than serous 17.1 (14.7–19.8) and endometrioid 13.1 (12–not estimable). A separate retrospective study of CCOC outcomes in Japan before and after the approval of first-line bevacizumab for advanced CCOC compared the survival of patients treated with carboplatin–taxane chemotherapy with patients who also received bevacizumab. This study reported an improved PFS from 12.5 to 29.7 months (*p* = 0.023) with the addition of bevacizumab, with multivariate analysis showing bevacizumab use was an independent prognostic factor for PFS (*p* = 0.011) and OS (*p* = 0.019).^45^ While this is encouraging, the authors do note that patients with thrombus were excluded from the bevacizumab arm but not the chemotherapy only arm; given that thrombus is associated with a worse prognosis in CCOC^46^ this introduces selection bias favouring improved outcomes in the bevacizumab arm from the outset.

A phase II study (ENGOT-GYN1) investigated the benefit of nintedanib (a kinase inhibitor of VEGFR1-3, PDGFR-α and -β and FGFR1-3) in recurrent CCOC. The response rate was 2.1% with nintedanib and 0% with chemotherapy with no survival benefit in terms of PFS; however, the authors reported a disease control rate at 16 weeks of 23.4% with nintedanib (compared to 9.1% with chemotherapy; *p* = 0.0276), suggesting biological activity of nintedanib that could be augmented with a combination approach.^47^

### Immunotherapy-based approaches

KEYNOTE-100 was a phase II open label multicentre study of the efficacy and safety of the anti-PD-1 antibody pembrolizumab in patients with advanced recurrent EOC. This study contained 19 patients CCOC with an overall response rate 15.8% (3.4–39.6 months) which was higher than other histological subtypes.^48^ Subsequent to this, a number of CCOC-specific studies have explored the role of checkpoint inhibition as a treatment for this cancer following first-line chemotherapy, as shown in Table 1.

**Table 1.** Recently published sub-type-specific clinical trials exploring novel therapeutic approaches in the treatment of CCOC, LGSOC and OCS.

Histological type	Trial agents	Phase	Primary endpoint	Comments	Trial ID
CCOC	Nintedanib *versus* chemotherapy	II	PFS	• mPFS 2.3 months nintedanib *versus* 1.9 months with chemotherapy • ORR was 2.1% with nintedanib *versus* 0% with chemotherapy • mOS 9 months nintedanib *versus* 4.9 months with chemotherapy	NCT02866370
	Pembrolizumab	II	PFS at 12 weeks	• PFS at 12 weeks was 43.8% • ORR 25% • mOS 71 weeks	NCT03425565
	Durvalumab *versus* chemotherapy	II	PFS	• mPFS 7.4 weeks durvalumab *versus* 14 weeks with chemotherapy • ORR 10.7% durvalumab *versus* 18.8% chemotherapy	NCT03405454
	Nivolumab and ipilimumab	II	PFS	• mPFS 2.7 months nivolumab *versus* 5.1 months with combination • ORR 14.2% nivolumab *versus* 26.7% with combination	NCT03355976
	PD1 and bevacizumab	II	ORR	*Preliminary results following enrolment of 23 of planned 38 patients;* • ORR 40%	NCT04735861
LGSOC	Ribociclib and letrozole	II	PFS at 12 weeks	Of three LGSOC patients included in this study, all responded to treatment (1 complete, 2 partial) with responses lasting over 2 years	NCT02657928
	Binimetinib *versus* chemotherapy	III	PFS	• mPFS 9.1 months with binimetinib *versus* 10.6 months with chemotherapy • ORR 16% binimetinib *versus* 13% chemotherapy • mOS 25.3 months binimetinib *versus* 20.8 months	NCT01849874
	Trametinib *versus* Chemotherapy	II/III	PFS	• mPFS 13 months trametinib *versus* 7.2 months with chemotherapy	NCT02101788
OCS	Paclitaxel with either carboplatin (TC) or ifosfamide (TI)	III	OS	• OS 30 months TC *versus* 25 months TI • mPFS 15 months TC *versus* 10 months TI	NCT00954174

This table provides an overview of some of the recent key trials in less common epithelial ovarian cancers. The trial agents are listed alongside the primary endpoint, key outcomes and trial identifier for clinicaltrials.gov.

CCOC, clear-cell ovarian cancer; LGSOC, low-grade serous ovarian cancer; mOS, median overall survival; mPFS, median progression-free survival; PD1, programmed death receptor 1; OCS, ovarian carcinosarcoma; ORR, overall response rate.

PEACOCC was a phase II study exploring treatment of clear-cell gynaecological malignancies with pembrolizumab, with the majority of patients having an ovarian primary. This study reported an overall response rate of 25% and disease control rate of 45.8% with median overall survival of 71 weeks.^49^ BrUOG 354 explored the activity of the PD-1 inhibitor nivolumab with and without ipilimumab (anti-CTLA4) in ovarian or extra-renal clear-cell pathologies; the stage 1 results of this study reported an overall response rate of 26.6% (*n* = 4/15) in the combination arm compared to 16.7% (*n* = 2/12) for nivolumab alone and so stage 2 will focus on dual treatment with nivolumab and ipilimumab.^50^ In comparison to these two studies, MOCCA was a phase II study exploring the role of the PD-L1 inhibitor durvalumab against physicians choice of chemotherapy; this study was negative with an overall response rate to durvalumab of 10.7% and no significant difference in OS.^51^ INOVA is a phase II study that is currently exploring the use of sintilimab (anti-PD1) in combination with bevacizumab in the treatment of recurrent CCOC; although this trial is still ongoing, preliminary results following the recruitment of 20 evaluable patients (out of a planned accrual of 38) have reported an overall response rate of 40%.^52^ As shown in Table 2, a number of other studies are also currently ongoing exploring the role of checkpoint inhibition in CCOC, the results of which are awaited.

**Table 2.** Ongoing clinical trials in the treatment of rare epithelial ovarian cancers.

Histological type	Trial agents	Mechanism of action	Phase	Trial ID
CCOC	AZD6738 and olaparib	ATR inhibitor and PARP inhibitor	II	NCT04065269
	Nivolumab and ipilimumab	PD1 inhibitor and CTLA4 inhibitor	II	NCT02834013
	Pembrolizumab and lenvatinib	PD1 inhibitor and VEGFR1-3 inhibitor	II	NCT04699071
	Sintilimab and bevacizumab	PD1 inhibitor and VEGFR inhibitor	II	NCT04735861
	Durvalumab and tremelimumab	PDL1 inhibitor and CTLA4 inhibitor	II	NCT02879162
LGSOC	Ribociclib and letrozole	CD4/6 inhibitor and aromatase inhibitor	II	NCT03673124
	VS-6766 and defactinib	RAF/MEK inhibitor and FAK inhibitor	II	NCT04625270
OCS	Niraparib and dostarlimab	PARP inhibitor and PD1 inhibitor	II/III	NCT03651206
	Cabozantinib, nivolumab and ipilimumab	TKI inhibitor, PD1 inhibitor and CTLA4 inhibitor	II	NCT04149275
All rare EOC	Multiple therapeutic agents	Biomarker-driven selection of agent	II	NCT04931342

This table details a number of ongoing clinical trials exploring novel therapeutic approaches in the treatment of rarer epithelial ovarian cancers.

The trial agents are listed alongside their mechanism of action and trial identifier for clinicaltrials.gov.

CCOC, clear-cell ovarian cancer; CTLA4, cytotoxic T-lymphocyte-associated antigen 4; EOC, epithelial ovarian cancer; LGSOC, low-grade serous ovarian cancer; PARP, poly-ADP ribose polymerase; PD1, programmed death receptor 1; PDL1, programmed cell death ligand 1; OCS, ovarian carcinosarcoma; VEGFR, vascular endothelial growth factor receptor.

The early results of these immunotherapy studies are encouraging and the focus will now be on identifying biomarkers which can help select which patients are more or less likely to benefit from checkpoint inhibition. The difference in the outcomes between PEACOCC and BrUOG when compared to MOCCA may be down to the differences in PD-1 ligand expression and the investigator choice of checkpoint inhibitor, with CCOC having high levels of PD-L2 expression^53^ but low or absent PD-L1^53–55^ which may have resulted in MOCCA targeting a less critical part of the PD-1 axis; however, the translational analysis of all three studies is awaited. Of the potential candidate markers, ARID1A is particularly of note. ARID1A has been linked to the effective functioning of the mismatch repair protein MSH2^56^ and when compared to ARID1A wildtype, ARID1A mutant CCOC had significant differences in immune cell populations, cytokine and checkpoint expression, many of which are predictive of response to checkpoint inhibition in other malignancies.^53^

## Mucinous ovarian cancer

Mutations in KRAS are the most common observed mutation in MOC, occurring in up to 65% of cases,^57^ with some evidence of racial variation impacting incidence.^58^ There are mixed reports regarding the mutual exclusivity of KRAS and human epidermal growth factor receptor 2 (HER2) aberrations in MOC.^57,59^ HER2 amplifications are observed in 35% of MOC,^60^ although as with KRAS mutation, the incidence may vary racially.^58,61^ Mutations in c-MYC are also common in MOC, with a reported incidence of up to 65%.^62^ The protein c-MYC impacts a wide variety of cellular pathways and in non-transformed cells levels are typically low.^63^

Similar to other EOCs, TP53 mutations are common in MOC, occurring in up to 57% of cases.^64^ Mutations in CDKN2A occur in up to 18.9% of MOC^57^ and encodes for two different proteins – p16 and p14ARF.^65^ Both these genes play essential roles in protecting against genomic instability by facilitating normal function of the cell cycle checkpoints. The protein encoded for by TP53 (p53) is key in the regulation of the G1 checkpoint and tumours with a TP53 mutation become reliant on the G2 checkpoint. Normally, the levels of p53 are kept low *via* a negative feedback loop with murine double minute 2 (MDM2); p14ARF helps abrogate MDM2 inhibition and facilitates the action of p53.^65,66^ Wildtype p16 arrests cells in late G1 and loss of normal p16 function results in the inability to induce cell-cycle arrest.^67^

### Molecular-guided therapies

MOCs have a similar mutational pattern to mucinous colorectal carcinomas^68^ with histological similarities resulting in metastatic deposits from other gastrointestinal mucinous carcinomas being frequently mistaken for primary MOC.^69^ This observation lay behind GOG0241, a phase III study which aimed to explore the use of colorectal chemotherapy regimens in the first-line treatment of MOC.^18^ Unfortunately, this study closed early due to slow accrual, illustrating the difficultly of undertaking large studies in this rare subtype. In the absence of large phase III studies, potential therapeutic approaches can be extrapolated from smaller studies of MOC alongside trials in malignancies with a similar genetic landscape, such as colorectal carcinoma.

KRAS was previously considered an undruggable target; however, the success of agents such as sotorasib^70^ and adagrasib^71^ (small molecules that selectively and irreversibly inhibit the G12C KRAS mutation) have demonstrated that for a subset of patients this is no longer the case. Ongoing clinical trials such as CodeBreak300^72^ and KRYSTAL-10^73^ are now exploring these drugs in the treatment of metastatic colorectal cancer. It remains to be tested whether this treatment is effective in a subset of KRAS mutant MOC, but this may merit further evaluation.

There are case reports of HER2 amplified MOC responding following treatment with trastuzumab (a HER2 targeting monoclonal antibody) alone^74^ or in combination with lapatinib (a tyrosine kinase inhibitor targeting HER2 and the epithelial growth factor receptor).^75^

Pre-clinical work in MYC amplified oesophageal cancer cell lines demonstrating a sensitivity to the BTK/HER2 inhibitor ibrutinib.^76^ Leading on from this, ibrutinib is currently being evaluated in the treatment of c-MYC and HER2 amplified gastro-oesophageal cancer.^77^ An alternative therapeutic approach targeting c-MYC mutant malignancies is to reduce the levels of the c-MYC protein, with a phase I study currently evaluating WB100, an oral molecule glue that pre-clinically selectively degraded c-MYC protein over other proteins.^78,79^

Wee1 is a tyrosine kinase essential to the successful execution of the G2 checkpoint and by inhibiting it, cells dependant on this checkpoint (such as those with a TP53 mutation) become sensitized to DNA damage.^80^ Adavosertib, a Wee1 inhibitor, is currently being explored in combination with chemotherapy for the treatment of platinum-resistant EOC. The interim analysis of this study has reported an overall response rate of 67% with mPFS of 10.1 months when adavosertib was given in combination with carboplatin^81^ with the final results awaited. Aurora kinases are essential for many aspects of cell division and essential for the normal transition into the G2 checkpoint.^82,83^ Ilorasertib is an inhibitor of aurora kinases (alongside VEGFRs and PDGFRs) and has been used in clinical trials for tumours with are CDKN2A deficient, the results of which are awaited.^84,85^

### Immunotherapy-based approaches

Despite a reported incidence of mismatch repair deficiency (MMRd) of 17%, over twice that of HGSOC,^86^ patients with MOC were excluded from the KEYNOTE100 trial.^48^ Patients with MMRd colorectal cancers who received pembrolizumab within the KEYNOTE-177 study were found to have an improved PFS when compared to chemotherapy^87^ and given that pembrolizumab has tissue/site-agnostic approval for MMRd tumours, checkpoint inhibition remains an option for a subset of MOC patients. Mismatch repair deficiency however remains only one predictor of response to checkpoint inhibition, with studies in urothelial cancer suggesting that harbouring a CDKN2A mutation reduces benefit from checkpoint inhibition, although this may be tumour specific as a second study in melanoma found no link between either CDKN2A or TP53 and clinical outcome following immunotherapy.^88,89^

## Low-grade serous ovarian cancer

Both oestrogen and progesterone are known drivers in the tumorigenesis of LGSOC, with up to 85% of cases oestrogen/progesterone receptor positive.^90^ As such, hormonal therapy has found a role in the treatment of patients with this malignancy either in favour of chemotherapy as a first line treatment,^91^ as a maintenance therapy following first-line chemotherapy^92^ or in the recurrent setting.^93^ Cyclin-dependant kinases 4 and 6 (CDK4/6) promote resistance to hormonal treatment in breast cancer^94^ with the combination of ribociclib (a CDK4/6 inhibitor) plus hormone treatment (letrozole) generating durable responses in all three patients with LGSOC given this combination within a phase II study.^95^ This combination is currently being explored as a treatment for LGSOC in an ongoing phase II study.^96^

Mutations in the RAS/RAF pathway occur in 47% of patients LGSOC.^90^ The MILO/ENGOT-ov11 study which investigated inhibition of this pathway using binimetinib (a MEK1/MEK2 inhibitor) compared to investigators choice of treatment demonstrated activity of binimetinib but did not demonstrate superiority to chemotherapy and was therefore stopped early due to the prespecified futility boundary.^97^ A second study, LOGS, explored the use of the MEK1/MEK2 inhibitor trametinib compared to investigators choice of treatment and demonstrated a significant improvement in PFS from 7.2 to 13 months (*p* ⩽ 0.0001), heralding a new standard of care option for patients with this histology.^98^ Building on this success, other studies are currently exploring treatments exploiting this pathway in LGSOC, such as RAMP-201, a study of the dual RAF/MEK inhibitor VS-6766 alone or in combination with the FAK inhibitor defactinib.^99^

## Ovarian carcinosarcoma

Due to the rarity of this tumour, information on the tumour microenvironment tends to be inferred from translational work performed on the relatively more common uterine and endometrial carcinosarcomas. The duality of its histological composition makes it additionally challenging to design personalized trials.^100^

### Chemotherapy

While the GOG0241 study demonstrated the clinical activity of cisplatin in the treatment of OCS^19^ and more recent translational work found platinum-containing agents were associated with a prolonged OS in OCS,^14^ the phase III study GOG261 failed to demonstrate the superiority of a platinum containing regimen. Although patients treated with paclitaxel–carboplatin had a longer overall survival (30 months *versus* 25 months) and PFS (15 months *versus* 10 months) when compared to paclitaxel–ifosfamide, these differences were not statistically significant.^101^ A high glucocorticoid receptor (GR) expression can identify patients who are less likely to derive benefit from nab-paclitaxel^102^ and given the high expression of GR in OCS,^103^ it may have a role as a biomarker for this patient population. These high GR levels may also mean that the GR modulator relacorilant could be beneficial in the treatment of OSC patients with recurrent disease.

### Immunotherapy-based approaches

Although OCS demonstrate a copy number high phenotype in 15/17 cases,^104^ PD-L1 expression and CD8+ T-cell infiltration are a feature of this disease,^105^ with whole exome sequencing of a single OCS sample demonstrating the presence of multiple tumour specific neo-antigens.^106^ Within the CA209-538 study, one PR and one SD were reported in five patients with OCS who received treatment with nivolumab and ipilimumab^107^ with an additional case report of a PR in recurrent OCS following treatment with pembrolizumab.^108^

Evidence of HRD was found in 15/25 patients with OCS^109^ with case reports of durable response to PARP inhibition.^110,111^ The ROSCAN phase II/III (NCT03651206) study will explore the role of PARP and checkpoint inhibition in the treatment of OCS using niraparib with and without concurrent dostarlimab (anti-PD1) in recurrent OCS.^112^ A second study is combining dual checkpoint blockade in the form of ipilimumab and nivolumab alongside the tyrosine kinase receptor inhibitor cabozantinib^113^; the use of cabozantinib has primary been in carcinosarcomas originating from other gynaecological sites^114^; however, VEGFR overexpression is reported 44% of OCS,^115^ indicating a rationale for this combination in OCS.

## Conclusions

Given the rarity of these tumours, particularly at an advanced stage, delivering high-quality phase III studies can be difficult, as illustrated by the GOG0241 study. A collaborative mobilization of patients *via* multi-centre, international studies is required if we are to improve patient outcomes. One example of this is the BOUQUET study (NCT04931342); a phase II basket study evaluating biomarker driven therapies in patients with rare EOCs, such as CCOC, MOC and LGSOC.^116^ Although to date HGSOC and HGEOC remain the only two EOC histologies to have a molecularly guided, personalized approach to systemic treatment, there is encouraging signs that other subtypes are moving this direction.

## Declarations

**Ethics approval and consent to participate:** Not applicable.

**Consent for publication:** Not applicable.

**Availability of data and materials:** Not applicable.
